# Economic impact of reducing treatment gaps in depression

**DOI:** 10.1192/j.eurpsy.2023.2415

**Published:** 2023-06-13

**Authors:** Paul McCrone, Allan H. Young, Roland Zahn, Jonas Eberhard, Danuta Wasserman, Paolo Brambilla, Judit Balazs, Jose Caldas-de-Almeida, Andrea Ulrichsen, Vladmir Carli, Ana Antunes, Giandomenico Schiena, Vinciane Quoidbach, Patrice Boyer, Rebecca Strawbridge

**Affiliations:** 1 Institute for Lifecourse Development, University of Greenwich, London, UK; 2 Department of Psychological Medicine, Institute of Psychiatry, Psychology and Neuroscience, King’s College London, London, UK; 3 South London and Maudsley NHS Foundation Trust, Bethlem Royal Hospital, Beckenham, UK; 4 Division of Psychiatry, Department of Clinical Sciences, Lund University, Lund, Sweden; 5 National Centre for Suicide Research and Prevention of Mental Ill-Health, Karolinska Institutet, Stockholm, Sweden; 6 Department of Neurosciences and Mental Health, Fondazione IRCCS Ca’ Granda Ospedale Maggiore Policlinico, Milan, Italy; 7 Department of Pathophysiology and Transplantation, University of Milan, Milan, Italy; 8 Department of Developmental & Clinical Child Psychology, Institute of Psychology, Eötvös Loránd University, Budapest, Hungary; 9 Department of Psychology, Oslo New University College, Oslo, Norway; 10 Chronic Diseases Research Center, Nova Medical School, Nova University of Lisbon, Lisbon, Portugal; 11 European Brain Council, Brussels, Belgium

**Keywords:** cost, depression, economics, treatment, gaps

## Abstract

**Background:**

Major depressive disorder (MDD) is highly prevalent across Europe. While evidence-based treatments exist, many people with MDD have their condition undetected and/or untreated. This study aimed to assess the cost-effectiveness of reducing treatment gaps using a modeling approach.

**Methods:**

A decision-tree model covering a 27-month time horizon was used. This followed a care pathway where MDD could be detected or not, and where different forms of treatment could be provided. Expected costs pertaining to Germany, Hungary, Italy, Portugal, Sweden, and the UK were calculated and quality-adjusted life years (QALYs) were estimated. The incremental costs per QALY of reducing detection and treatment gaps were estimated.

**Results:**

The expected costs with a detection gap of 69% and treatment gap of 50% were €1236 in Germany, €476 in Hungary, €1413 in Italy, €938 in Portugal, €2093 in Sweden, and €1496 in the UK. The incremental costs per QALY of reducing the detection gap to 50% ranged from €2429 in Hungary to €10,686 in Sweden. The figures for reducing the treatment gap to 25% ranged from €3146 in Hungary to €13,843 in Sweden.

**Conclusions:**

Reducing detection and treatment gaps, and maintaining current patterns of care, is likely to increase healthcare costs in the short term. However, outcomes are improved, and reducing these gaps to 50 and 25%, respectively, appears to be a cost-effective use of resources.

## Introduction

Major depressive disorder (MDD) is highly prevalent across the world. Within Europe the prevalence of current MDD has recently been estimated at 6.38%, ranging from 2.58% in the Czech Republic to 10.33% in Iceland [1]. It has been suggested that recovery from a single episode is experienced by about half of the patients, with an unremitting course for 15% and recurrent illness for 35% [2]. MDD imposes high economic costs in terms of the use of services and its impact on employment [3].

There is a good evidence base to support the use of medication and different types of psychological therapy in the treatment of MDD [4]. However, it is well-known that many people are resistant to treatment, [5, 6] and that there are gaps in both detecting MDD and the provision or uptake of effective treatment and continuity of care [7]. Some people who experience an episode of MDD may go on to have a natural recovery in the absence of treatment and so it is unclear whether removing all treatment gaps is necessary. In economic terms there may well be diminishing returns in doing so.

Reducing or removing these treatment gaps will have an impact on healthcare costs. Although this is somewhat complex as more provision or uptake will increase direct care costs, these may be offset by reduced costs elsewhere in the system (for example, less need for subsequent crisis support or even the use of physical health services). We would also hope to see a positive impact on clinical outcomes because of reduced treatment gaps and this would hopefully lead to improved health-related quality of life. It may also result in broader beneficial impacts on work, education, and social participation [8].

Providing or increasing services for people with MDD, as with any other condition, requires the use of resources that could be used in alternative ways. The coronavirus disease-2029 (COVID-19) pandemic has shown that when there is political will, resources allocated to healthcare can be substantially increased. However, the normal state of affairs is that governments or other providers and funders prefer to operate within constrained budgets, and as such resource scarcity is imposed. Given this, it is necessary to assess new therapies and services or increase their provision of them in terms of cost-effectiveness. This then allows decision-makers to choose how best to allocate resources between competing alternatives.

Cost-effectiveness can be assessed using data collected within a trial or through simulation modeling. Trials allow for reliable comparisons to be made between treatment options and have high internal validity, although they are expensive and time-consuming, and findings may not be generalizable to other settings. Models are a way of focusing on the most salient aspects of the care process and results can be derived more quickly. They are a simplification of real life and uncertainty around model parameters can be high. As such, it is important to subject models to extensive sensitivity analyses to see how robust findings are. Modeling approaches have been used previously by us in evaluations of mental health services [8–10].

This paper seeks to model the economic impact of reducing treatment gaps for people with MDD in six European countries: Germany, Hungary, Italy, Portugal, Sweden, and the UK. It aims to (i) compare the costs of providing care in each country to reduce the treatment gaps and (ii) compare the cost-effectiveness in terms of increased quality-adjusted life years (QALYs) between countries for reducing the treatment gap.

## Methods

The analyses presented in this paper are part of the Value of Treatment (VOT) program funded by the European Brain Council. The VOT program focuses on care provided to people with a number of neurological or mental health conditions including MDD and brings together experts including clinicians and health economists from across Europe.

### Model structure

**Figure 1.**
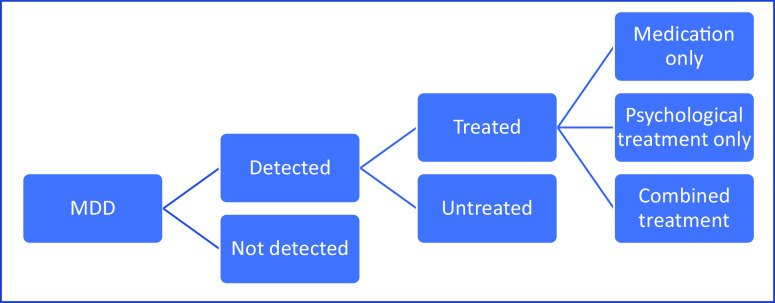
Decision model to assess economic impact of reducing treatment gaps (reduced model).

The process for modeling the cost-effectiveness of reducing treatment gaps first required the establishment of the model structure (Figure 1). The model follows the form of a decision tree and focuses on some key aspects of the care process. Patients with MDD either have their condition detected or not. If they do, they then receive medication only, psychological therapy (assumed to be cognitive behavioral therapy [CBT]) only, combined psychological therapy and medication, or else are untreated. A healthcare perspective is taken and the time horizon of the model is 27 months in line with the study of Koeser et al. [11] (This was chosen to provide a two-year follow-up subsequent to a three-month treatment phase.) An important point to emphasize is that this is not a model which is intended to compare the different treatment options themselves. The paper by Koeser et al. [11] did this and the findings from that work are used here.

### Model parameters

To run the models, we use data on the probabilities of different events occurring, the costs of those events, and the QALYs accrued over time which depend on treatment outcomes. This allows us to estimate expected costs and QALYs with existing patterns of care and then following the reduction of treatment gaps.

Probabilities for the model are obtained from a linked study [7], using data from Germany, Hungary, Italy, Portugal, Sweden, and the UK. From the countries included in this linked study, we estimate that 69% of cases of MDD are undetected [7]. Of detected cases, 50% are assumed to receive treatment and of these we estimate 70% receive medication only, 23% receive psychological therapy, and 7% receive both. The costs of care and associated QALYs are based on recognized therapies (medication, CBT, or combined treatment). These costs are from Koeser et al. [11], and take into account differences between the therapies in terms of remission and relapse. The Kuyken et al. [12] study obtained healthcare cost data from previous work and the services included primary and secondary healthcare contacts. It was a UK study and the services are usually government financed. Koeser et al. [11] discounted the costs and QALYs at a rate of 3.5% per annum. Given the costs are from the UK we make appropriate adjustments based on purchasing power parities to reflect healthcare costs in Germany, Hungary, Italy, Portugal, and Sweden [13]. The set of parameters used to run the base case model is detailed in Table 2. Assumptions needed to be made about costs and QALYs for undetected and untreated cases. For QALYs, it was assumed that these would be half of those achieved by medication alone (i.e., 0.618). For costs, we arbitrarily assumed a figure of €1000 for untreated cases and €750 for undetected cases.

**Table 1.** Model parameter values

Parameter	Base case value	References
*Probabilities*		
Depression detected	0.31	Strawbridge et al. [7]
Depression undetected	0.69	Strawbridge et al. [7]
Detected cases receive medication only	0.70	Strawbridge et al. [7]
Detected cases receive psychological therapy only	0.23	Strawbridge et al. [7]
Detected cases receive combination therapy	0.07	Strawbridge et al. [7]
Detected cases are untreated	0.50	Strawbridge et al. [7]
*Healthcare costs for 27-month period (2020 Euros)*		
Medication therapy	4,930	Koeser et al. [11]
Psychological therapy	5,975	Koeser et al. [11]
Combination therapy	6,843	Koeser et al. [11]
Undetected cases	750	Estimate
Untreated cases	1,000	Estimate
*Quality adjusted life years for 27-month period*		
Medication therapy	1.236	Koeser et al. [11]
Psychological therapy	1.274	Koeser et al. [11]
Combination therapy	1.274	Koeser et al. [11]
Undetected cases	0.618	Estimate
Untreated cases	0.618	Estimate

*Note*: Multipliers to convert UK costs to those in other countries: Sweden 1.4, Portugal 0.63, Germany 0.83, Italy 0.95, and Hungary 0.32 [13].

**Table 2.** Incremental costs, QALYs, and ICERs of reducing detection and treatment gaps

	Germany	Hungary	Italy	Portugal	Sweden	UK
Base case costs	1,236	476	1,413	938	2,093	1,495
Base case QALYs	0.716	0.716	0.716	0.716	0.716	0.716
*Detection gap reduced to 50%*						
Incremental costs	378	145	432	286	639	456
Incremental QALYs	0.0598	0.0598	0.0598	0.0598	0.0598	0.0598
ICER	6,315	2,429	7,217	4,788	10,686	7,633
*Treatment gap reduced to 25%*						
Incremental costs	399	153	456	303	675	482
Incremental QALYs	0.0488	0.0488	0.0488	0.0488	0.0488	0.0488
ICER	8,180	3,146	9,349	6,203	13,843	9,888

*Detection gap reduced to 50% and treatment gap to 25%*						
Incremental costs	822	316	940	624	1392	994
Incremental QALYs	0.1385	0.1385	0.1385	0.1385	0.1385	0.1385
ICER	5,941	2,285	6,790	4,505	10,054	7,182

### Analysis

Running the model with the data contained in Table 1 allowed us to calculate the expected costs and QALYs for the base case. We then made adjustments to the probabilities of detection and treatment being provided and recompute the expected costs and expected QALYs. This then allowed us to calculate incremental cost-effectiveness ratios (ICERs) by dividing the incremental costs by the incremental QALYs.

Uncertainty around the values of model parameters was addressed using a series of one-way sensitivity analyses. Uncertainty particularly exists around the costs for cases of undetected and untreated MDD and the QALYs that accrue for these cases. These costs were initially assumed to be €750 and €1000, respectively, in the UK and sensitivity analyses were conducted whereby these were increased or decreased by 25%. In addition, rather than assume that those with undetected and untreated MDD have outcomes that are only half as good as those detected and treated with medication, we instead assume that they do three-quarters as well. This reflects the possibility that natural recovery or improvement may occur for many even in the absence of treatment. Finally, we have altered the distribution of treatment options by assuming that 50% of treated cases receive combination therapy, 23% still receive CBT alone, and 27% receive medication alone. The sensitivity analyses were applied just to the scenario where the detection gap is reduced to 50% and the treatment gap to 25%.

## Results

Expected costs and QALYs over a 27-month period for those with MDD based on the model estimates are shown in Table 2 for each of the six countries [7]. These costs reflect differences in healthcare prices across the countries with the highest costs in Sweden and the lowest in Hungary. Expected QALYs are 0.716 assuming that those undetected or untreated do half as well as those on medication.

**Figure 2.**
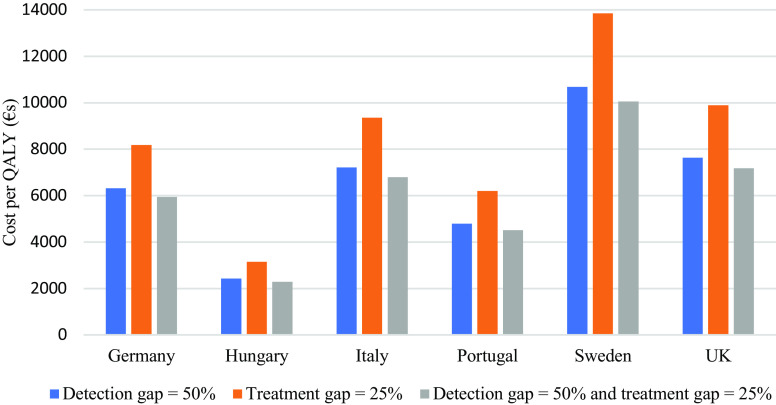
Incremental cost per QALY of reducing detection and treatment gaps.

Based on the model, we show that if the detection gap is reduced from 69% to 50% there are increased costs over a 27-month period but also increased QALYs (Table 2). This is also the case for a reduced treatment gap from 50% to 25% for cases that are detected. If both the detection gap and treatment gap are reduced, then both costs and QALYs increase more than in these other scenarios but the ICER is lower than for both gap reductions on their own. In the UK, the willingness-to-pay threshold is £20,000 per QALY (around €23,000) and it can be seen that each of these scenarios would be cost-effective with current models of care. If extra resources are required to reduce the detection and treatment gaps then we can see how much these could amount to and remain below the threshold. For example, in the UK the incremental cost associated with reducing the detection gap to 50% is €456. This could increase by €919 and cost-effectiveness would still be achieved. Similar findings apply to the other countries although QALY thresholds vary and do not always apply (Figure 2).

The findings from the sensitivity analyses are shown in Table 3. When the costs associated with MDD being undetected or untreated are decreased by 25%, the ICERs increase slightly but decrease if these costs are increased by 25%. This is logical as the sensitivity analyses are applied to the scenario where both detection and treatment gaps are reduced. The cost-effectiveness of doing this is going to be greater if not detecting or treating is associated with higher costs. The ICERs are increased if the distribution of treatment is changed to reflect more people receiving combination therapy. While the QALYs accrued from different treatments are similar, the costs are higher if more people receive combination therapy. The greatest impact on ICERs was made by changing the QALYs accrued for those who have their MDD undetected or untreated. If they do 75% as well as those treated with medication, then the ICERs are more than doubled. This is because in the absence of detection or treatment, outcomes are not as poor as in the original analyses and so gains from reducing gaps are less.

**Table 3.** Sensitivity analyses based on reducing detection gap to 50% and treatment gap to 25%

	ICERS compared to base case (2020 Euros)					
	Germany	Hungary	Italy	Portugal	Sweden	UK
No sensitivity analysis	5,941	2,285	6,790	4,505	10,054	7,182
Costs of undetected and untreated cases decreased by 25%	6,199	2,384	7,084	4,700	10,490	7,493
Costs of undetected and untreated cases increased by 25%	5,684	2,186	6,496	4,310	9,618	6,870
Combination therapy 50%, CBT 23%, medication 27%	6,845	2,633	7,823	5,190	11,583	8,274
QALYs for undetected and untreated cases 75% of those treated with medication	11,671	4,489	13,338	8,849	19,751	14,108

## Discussion

Reducing gaps in the detection and treatment of MDD is important for the quality of life of those affected. However, the analyses presented in this paper indicate that reducing treatment gaps will increase care costs. This is likely to be particularly the case in the short term. However, increased costs can be entirely justified if outcomes are sufficiently improved. The findings from our modeling work suggest that QALYs are increased if more cases of MDD can be detected and if more of those who do have MDD detected go on to receive treatment. The ICERs suggest that interventions to reduce the treatment gaps may represent good value for money. However, those interventions will themselves have costs and this would need to be offset against the estimates here. Increased training for non-specialists clinicians and use of paper-based or electronic screening instruments may be viable interventions.

The sensitivity analyses show that of crucial importance is the assumption made about the outcomes that occur for those who do not have their MDD detected or treated. In our initial analyses, we assumed that QALYs are half those that would occur if someone with MDD was treated with medication. When we assume that those with undetected or untreated MDD do 75% as well as those receiving medication then the cost-effectiveness of reducing detection and treatment gaps is much reduced. This is logical as it would imply that more people would recover naturally and so not as much is to be gained from reducing gaps. While studies have investigated the natural history of depression in the absence of treatment [14], there have been few studies that have examined the health-related quality of life for undetected or untreated cases of MDD.

There are limitations to the study. First, we have only focused on detection and treatment gaps and have not attempted to compare the different forms of treatment. The costs and outcomes from medication, psychological therapy, and combination therapy were fixed within the model and taken from one source [11]. That study did though conduct a thorough meta-analysis of treatment effectiveness and costs reported in other studies. Second, the data are mainly from the UK and although we made conversions to reflect costs in other countries this did assume that the structure of care was the same. Third, we have taken a healthcare perspective in our analyses. The main economic effect of improving care for people with MDD may come from outside of the healthcare sector, particularly in terms of employment [15]. Indirect costs of depression are often substantially higher than healthcare costs due to lost work [15]. Finally, we have not evaluated interventions that will actually result in reduced detection or treatment gaps. Further work on this is required but the analyses do suggest that they could be cost-effective if reasonably priced.

In conclusion, even when using conservative estimates of treatment effects and costs, reducing detection and treatment gaps for MDD is likely to be cost-effective across different European countries. If we also looked at indirect costs, then further benefits may be apparent that could potentially pay for interventions to reduce the gaps.
